# Supplementary material for: Acptp2,3 participates in the regulation of spore production, stress response, and pigments synthesis in Aspergillus cirstatus
Source: PeerJ. 2024 Sep 18;12:e17946. doi: 10.7717/peerj.17946 (PMC11416076; doi:10.7717/peerj.17946)
Supplement: Supplemental Information 3 — Primer information in this study. [file peerj-12-17946-s003.docx]

Table S1 Primers information in this study

| Primer | Sequences (5’-3’) | Length (bp) |
| --- | --- | --- |
| Up-*Acptp2,3*-F | CCGCTCGAGTCTGCCATGACAGGTCCGAG | 29 bp |
| Up-*Acptp2,3*-R | CGCGGATCCAATCACAGCCACCGATTGGC | 29 bp |
| Down-*Acptp2,3*-F | CGCGGATCCGTGTACTCGAATGGGTTGTTTCTCAGA | 36 bp |
| Down-*Acptp2,3*-R | GCTCTAGAGGTTTCAGCAAGTCTTGTTGGCT | 31 bp |
| Qc-*Acptp2,3*-F | GGGGTACCATGTGAACGGGAACCATTTGAAAGC | 33 bp |
| Qc-*Acptp2,3*-R | CCCAAGCTTTCTCGTAACATAGCACGAACTGACC | 34 bp |
| *Acptp2,3*-K-F | TCTGCCATGACAGGTCCGAG | 20 bp |
| *Acptp2,3*-K-R | TCTCGTAACATAGCACGAACTGACC | 25 bp |
| *RT-Acptp2,3-F* | GAACCATTTGAAAGCCGAATATA | 23 bp |
| *RT-Acptp2,3-R* | AAAAAGAAAGAAAAAAGGAGGAA | 23 bp |


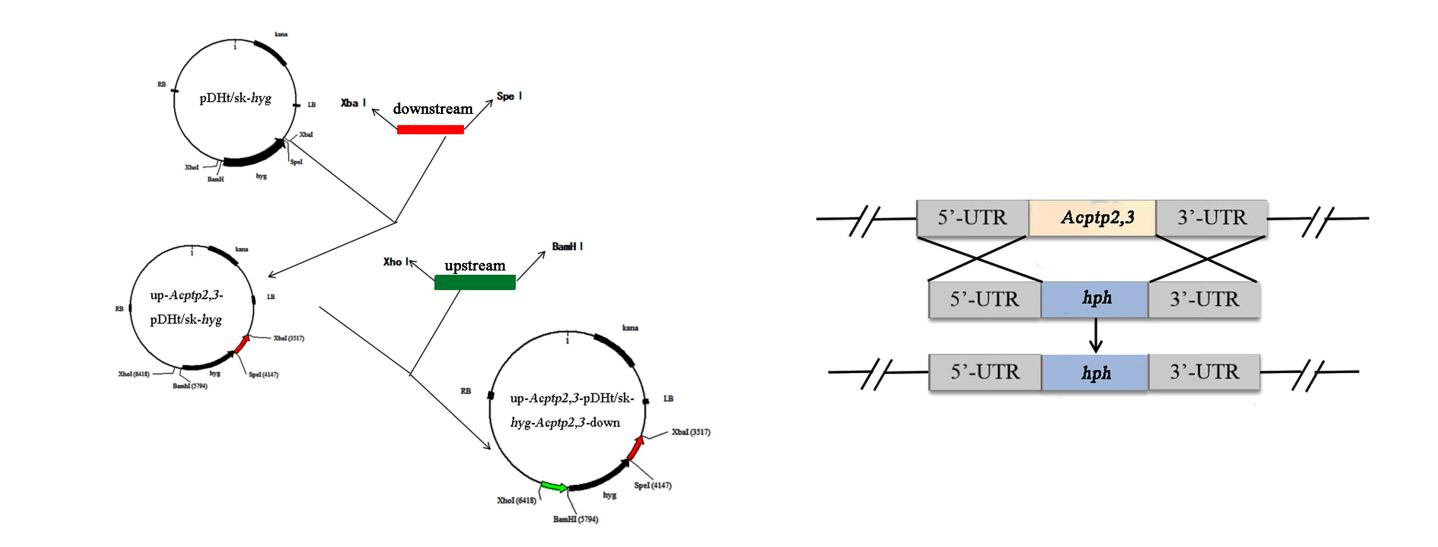


Figure S1 Schematic diagram of plasmid construction


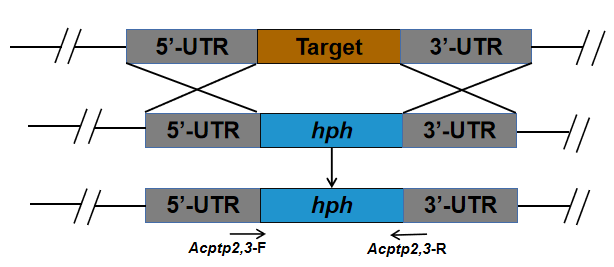


Figure S2 Primers designed for verification of Δ*Acptp2,3* deletion transformants


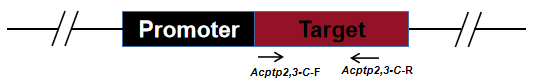


Figure S3 Primers designed for verification of Δ*Acptp2,3-C* complementary transformants


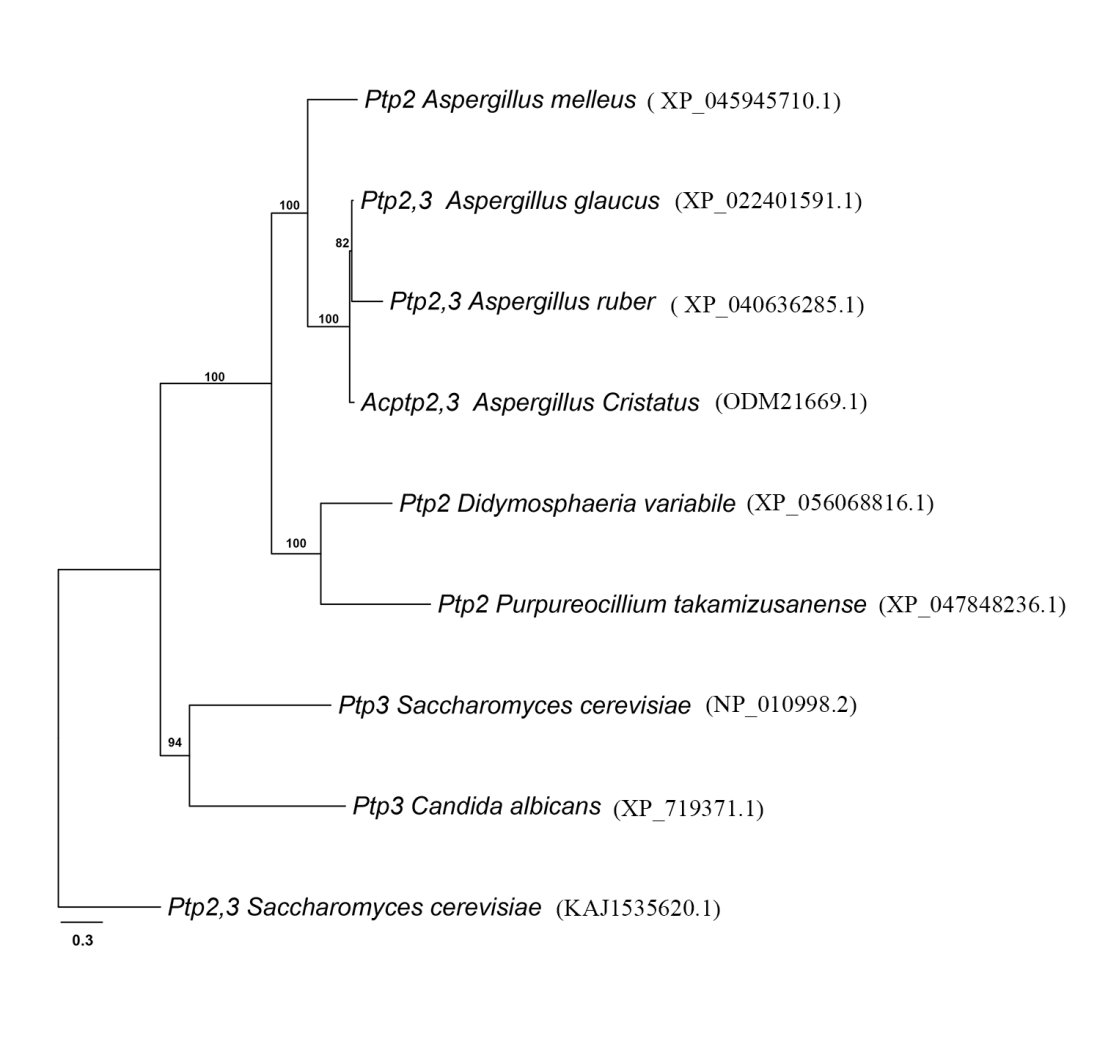


Figure S4 Homology study of Pbs2 proteins. A phylogenetic tree of Ptp2,3 in various fungi. MEGA 6.0 was used to create phylogenetic trees using the maximum likelihood method and a bootstrap value of 1000. The numbers above each node are the bootstrap values. The scale bars represent the amount of substitutions per amino acid site.


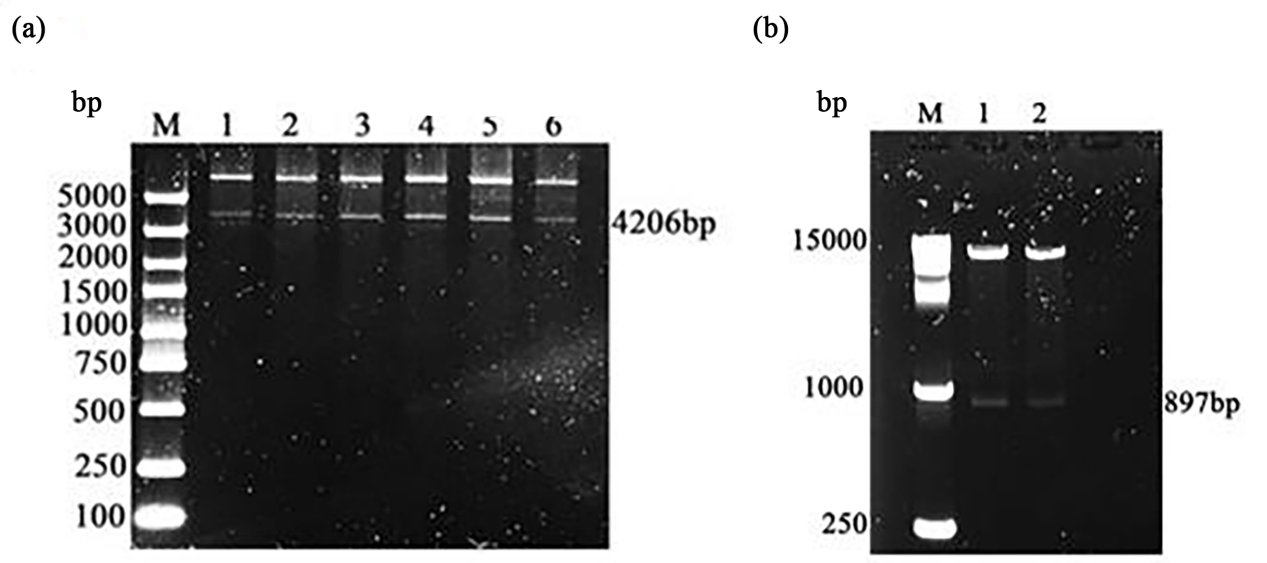


Figure S5 Validation of the up-*Acptp2,3*-pDHt/sk-*hyg*-*Acptp2,3*-down recombinant plasmid and *Acptp2,3* complementation plasmid. (A) *A. tumefaciens* transformants bearing recombinant plasmid up-*Acptp2,3*-pDHt/sk-*hyg*-*Acptp2,3*-down were validated using double digestion. M: 5000 marker; lanes 1–6: double digestion. Validation of *A. tumefaciens* transformants with recombinant plasmid up-*Acptp2,3*-pDHt/sk-*hyg*-*Acptp2,3*-down. (B) Verification of the *Acptp2,3* complementation vector construction. M: 15000 Marker; lanes 1-2: double enzyme digestion to verify the *Acptp2,3* complementation vector.


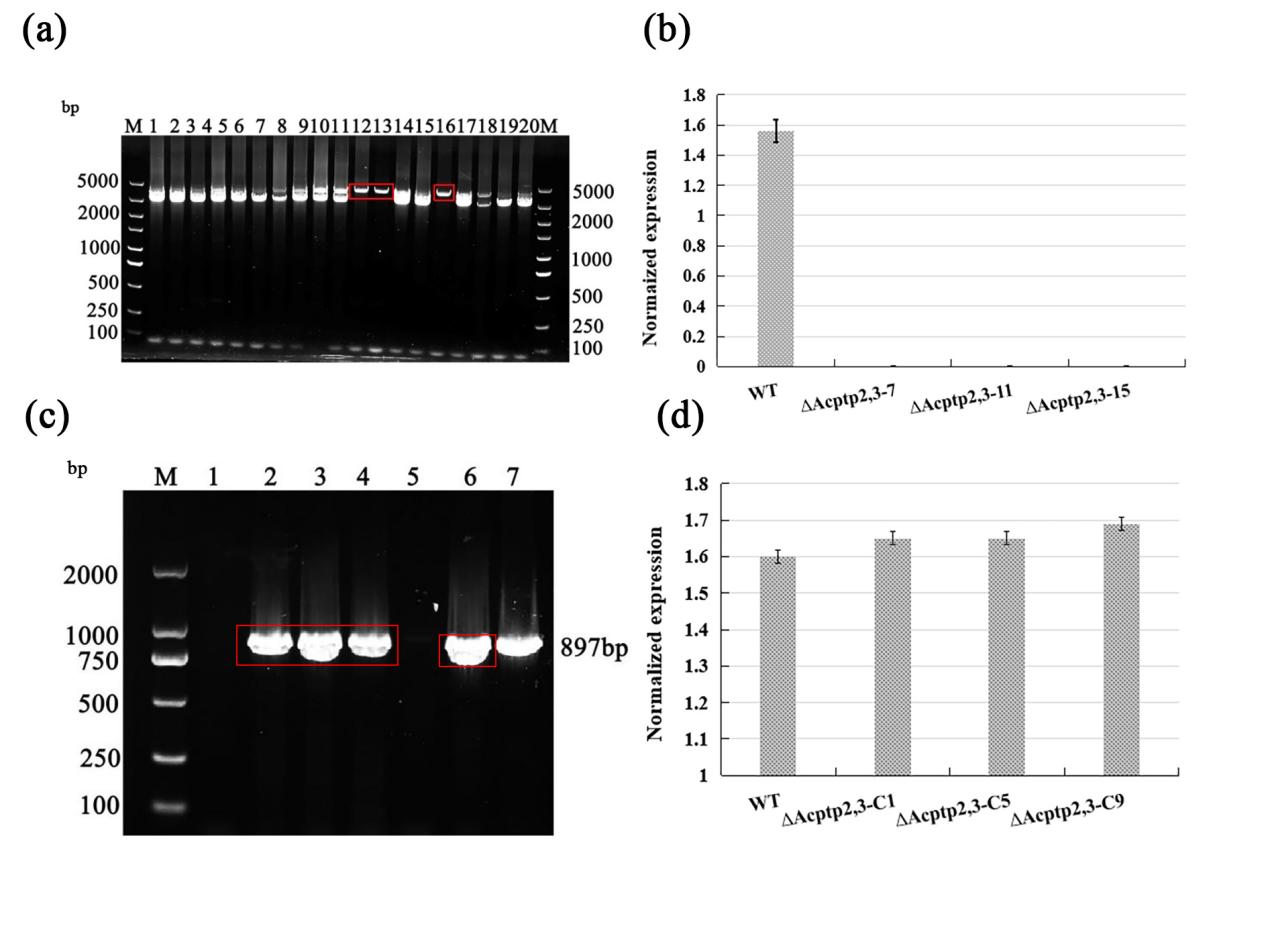


Figure S6 Verification of the Δ*Acptp2,3* and Δ*Acptp2,3*-C strains. (A) PCR verification for Δ*Acptp2,3*. The red boxes indicated the Δ*Acptp2,3* strains. (B) Measurement of *Acptp2,3* gene expression in knockout transformants. (C) PCR verification for Δ*Acptp2,3*‐C. Lane 7 was a wild-type control. (D) RT-qPCR detected *Acptp2,3* expression levels in Δ*Acptp2,3*-*C*. The data was normalized.
